# Student confidence in clinical competence during dental education

**DOI:** 10.1038/s41405-024-00274-9

**Published:** 2024-12-05

**Authors:** Malin Brundin, Mats Sjöström

**Affiliations:** 1 Division of Endodontics, Department of Odontology, Umeå University, Umeå, Sweden; 2 Division of Oral and Maxillofacial Surgery, Department of Odontology, Umeå University, Umeå, Sweden

**Keywords:** Dental clinical teaching, Dental education

## Abstract

**Introduction:**

Dental education blends theoretical and practical training to prepare students for independent patient care. This study examines the confidence levels of dental students at Umeå University, Sweden regarding independent performance of general dentistry tasks, and whether this confidence changes after clinical training.

**Material and methods:**

Surveys were conducted before and after a summer clinical internship, with students rating their comfort levels on a scale from 0 to 10 for various procedures.

**Results:**

Results from 64 initial surveys and 54 follow-up surveys showed no significant difference in confidence before and after the internship except for performing a tooth extraction (*p* = 0.010). However, confidence levels varied based on whether students had practiced specific tasks during the internship.

**Discussion:**

Students felt least confident in managing acute dental trauma (mean 6.1–6.2) and treating cavities in young children (mean 6.2–6.7), while they were most confident in tasks like obtaining radiographic surveys (mean 8.5–8.6) and debriding periodontally compromised dentition (mean 8.2–8.3). Tooth extraction and dental fillings were the most practiced procedures during the internship.

**Conclusions:**

The study concludes that the internship between the ninth and tenth semesters does not notably boost students’ confidence in specific dental procedures except for dental extractions. This result can possibly be explained due to the short duration of the training. Further exploration, including input from clinical supervisors, is suggested to optimise clinical training in dental education.

## Introduction

As a newly graduated dentist, skills are required to be able to handle the full spectrum of situations that arise within professional practice including issues related to the diagnosis and treatment of odontological conditions [1, 2] Dental education in Umeå, Sweden is a 5-year programme that encompasses theoretical and practical training designed to equip students with the skills to independently manage preventive, operative, and postoperative care in a patient-safe manner. The early years of the curriculum focus on foundational medical subjects and the later years focus on clinical training. The clinical training gradually intensifies in both duration and complexity, underscoring the pivotal role of strong knowledge and clinical foundations as students transition into the professional arena. At Umeå University, the manual training begins on plastic models in a simulated environment in the third semester. However, it has been shown that despite the significant improvement in digital educational tools in recent years, the best training is achieved when individuals are given the opportunity to perform the procedures on real subjects [3]. An integral part of the curriculum is to demonstrate professionalism, communication, and social skills [2], which are challenging to train in a simulated environment. The basic clinical training on patients is performed under supervision at the educational clinic and starts in the fifth term, where patients with diverse healthcare needs seek treatment. Guided by experienced dentists, students provide patient care, with case complexity increasing as they progress through the curriculum. After completing nine terms, students can independently practice general dentistry in public dental care during the summer break before returning to the dental school for their final term.

In addition to fostering a conducive learning environment, the student’s confidence and self-esteem are crucial factors in the educational process [4, 5]. These attributes facilitate knowledge acquisition and instil a sense of security in their clinical roles [6]. Confidence and positive attitudes toward learning empower individuals, motivating them to confront new challenges with a sense of competence [5]. Healthy self-esteem not only eases interactions with instructors but also encourages active participation, discussions, and questioning in various learning situations [7]. Conversely, low self-esteem or a lack of confidence may lead to self-doubt, hindering students from fully engaging in the learning process and developing clinically [8]. Confidence in clinical work not only influences students’ performance but also plays a significant role in shaping their professional identity and readiness for practice [9].

Understanding the dynamics between confidence and competence is crucial for educators and healthcare institutions aiming to optimise educational practices and enhance student outcomes.

This observational study investigates how confident students are in independently performing clinical tasks within general dentistry and whether their confidence in their clinical competence changes after completing their clinical training in general dentistry.

By shedding light on these questions, this study aims to contribute to the ongoing discourse surrounding student preparation for clinical practice. Ultimately, the findings of this research attempt to inform educational practices that empower students to navigate the complexities of clinical environments with confidence and proficiency, thus ensuring the delivery of optimal patient care. The null hypothesis is that there is no difference in students´ confidence in clinical competence after clinical training in general dentistry during summer break.

## Material and methods

In the fifth academic year of the dental education programme in Umeå, students were invited to respond to a survey with the aim of investigating their comfort level in the following clinical subjects: cariology, endodontics, clinical oral physiology, oral radiology, oral surgery, orthodontics, periodontology, paediatric dentistry, and prosthodontics. The students were informed that the survey would be conducted at the end of the ninth semester and after their summer clinical internship (i.e., at the beginning of their tenth semester). Participation in the surveys was voluntary. In conjunction with completing the surveys, informants provided their consent to participate in the study. The study underwent ethical review by the Swedish Ethical Review Authority. Because no intervention or other action would be performed on a research subject and no personal data would be processed, the Ethical Review Authority concluded that the study is not subject to the Swedish Ethical Review Act and therefore does not require ethical approval (Reference number: 2022-01934-01).

On 18 May 2022 (just before the summer break), students (*n* = 67) were given the opportunity to anonymously complete Survey 1, which consisted of rating 10 statements (one within each clinical subject). Each question was answered by making a mark on a 10-cm line, where 0 = ‘I feel absolutely uncomfortable/insecure performing the stated treatment procedure’ and 10 = ‘I feel completely comfortable/secure performing the stated treatment procedure’. This scale is often used in medicine to measure patients’ rating of pain intensity, but it is also used for measuring other subjective experiences, such as confidence [10–13].

The clinical procedures that the students assessed are listed in Table 1. The statements the students were asked to evaluate were formulated in consultation with the course coordinator for the various clinical subjects to ensure that students had received education covering the specific treatments mentioned in the survey.

**Table 1:** Statements within the various clinical subjects that students self-assessed before and after independently practicing general dentistry in public dental care between the ninth and tenth semesters in the dental programme.

Statement	Clinical subject
"After the training I have attended at the dental school, I feel knowledgeable enough to…	
…perform a tooth extraction"	Oral Surgery
…perform access preparation for endodontic treatment"	Endodontics
…prepare a tooth for a dental crown"	Prosthodontics
…excavate a tooth and perform a permanent filling"	Cariology
…restore a tooth in a young child (<6 years old)"	Paediatric Dentistry
…determine the position of the upper canines in the mixed dentition"	Orthodontics
…debride a periodontally compromised dentition"	Periodontology
…manage acute dental trauma"	Dental trauma
…perform a radiological full mouth survey"	Oral Radiology
…diagnose a patient with temporomandibular joint disorder (TMD)"	Clinical Oral Physiology

When the students returned to their education, 29 August 2022, they responded to the same survey with the addition of indicating whether they had performed the clinical moment during their summer clinical internship in public dentistry (Survey 2). The responses were recorded on two separate instances by two different individuals, and inter- and intra-reliability were calculated for their registrations. To test the difference in the students’ responses before and after training, the Mann-Whitney U-test was used. The significance level for all tests was set to 5%. All statistical analyses were conducted using IBM SPSS Statistics for Windows, Version 26.0 (Armonk, NY: IBM Corp).

## Results

In this study, all students (67 students; 46 females and 21 males) enroled in the ninth semester of the dental programme in Umeå were offered the opportunity to respond to surveys regarding their confidence levels in various dental procedures. The survey was administered on two occasions: 18 May 2022, right before the students’ summer break, and 29 August, the first day the students returned after the summer break. During the summer break, the students had independently worked in public general dentistry in Sweden.

Of the 67 students, 64 completed the survey before the summer break (Survey 1), and 54 completed the survey after the break (Survey 2). The mean values of the students’ responses are presented in Table 2. Of the ten clinical subjects addressed in Survey 1, students indicated feeling most uncertain about managing an acute dental trauma and restoring a cavity in a young child (<6 years old). They expressed the highest confidence in obtaining a radiographic full-mouth survey and debriding a periodontally compromised dentition. These findings were also reported in Survey 2.

**Table 2:** Students reported self-confidence before and after clinical internship.

Procedure	Before summer internship (*n* = 64)	After summer internship (*n* = 54)	*p*-value	Not performed the procedure during the internship		Had performed the procedure during the internship		*p*-value
	Mean	Mean		responders (*n*)	Mean	responders (*n*)	Mean	
Perform a tooth extraction	6.4	7.1	0.010	5	4.5	48	7.4	0.018
Perform an access preparation on a tooth	6.6	6.5	0.839	15	4.8	35	7.2	<0.001
Perform a crown preparation	8.1	7.4	0.060	21	7.2	14	7.7	0.583
Excavate and place direct restauration	8.4	8.7	0.328	1	9.3	53	8.7	–
Restore a tooth in a young child	6.2	6.7	0.181	15	4.9	25	7.7	0.003
Determine the position of the upper canines	7.9	7.4	0.189	17	6.6	29	7.8	0.050
Debride a periodontally compromised dentition	8.3	8.2	0.866	19	7.3	26	8.9	<0.001
Manage acute dental trauma	6.1	6.2	0.609	15	4.7	34	6.9	0.005
Perform a radiological full mouth survey	8.6	8.5	0.967	21	7.9	27	8.9	0.070
Diagnose a patient with TMD	8.3	8.0	0.447	10	7.5	40	8.1	0.409

In the survey administered after the students worked in general dentistry (Survey 2), they were also asked to report whether they had the opportunity to perform the various clinical tasks during their summer clinical internship (Table 2). The procedures most practiced during the internship were tooth extraction and dental fillings. The tasks that most students did not perform during their internship were crown preparation and obtaining a radiographic full mouth survey.

**Fig. 1:**
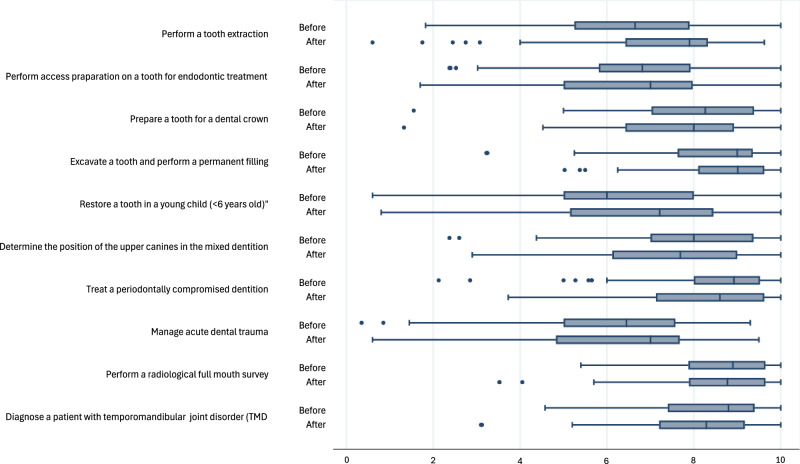
Differences in self-confidence before and after clinical internship.

The students reported a significant increased self-confidence (*p* = 0.010) concering the statment ”*performing a tooth extraction”* after the summer clinical internship (Fig. 1 and Table 2). No other significant differences in self-confidence were seen before and after the summer clinical internship. In the comparacy between students performing the clincial tasks to students not performing the clinical tasks, significant differences were found in statement number 1, 2, 5, 7 and 8 in reported self-confidence (Table 2).

## Discussion

The findings of this assessment, examining the self-confidence of Umeå dental students before and after their engagement in public dentistry beyond the academic setting, suggest that there was no overall change in dental students’ self-confidence. Our null hypothesis could only be rejected for “*performing a tooth extraction* “, while the null hypothesis for the other nine clinical tasks could not be rejected as no differences in self-confidence before and after clinical internship was found.

In this study, there is no information regarding the amount of time students worked in general dentistry during their 12-week summer break. Thus, we do not have information on the number of patients the students treated during this period. Since we aimed to examine the students’ confidence before and after they were allowed to work temporarily authorised during the summer, we were restricted to the number of students enrolled in the course. All students were invited to participate in the study, and all but three accepted. However, we had a 15% dropout rate, as only 54 of the invited students completed the second survey. Since the survey was anonymous, we were unable to assess the reasons for the dropout, which is a limitation. This situation with anonymity made also test-retest for the individual student impossible.

The clinical procedures evaluated in this study are representative of tasks routinely encountered in everyday dental practice and are commonly performed by dentists. Therefore, the nature of these procedures does not require an extended period of clinical work to ensure exposure to the range of tasks. This consideration is relevant, as the students’ opportunity to work in general dentistry was limited to a 12-week period. One might speculate whether the number of treated patients was insufficient to yield improvements in clinical experience. At the end of the ninth semester, the students had received relatively comprehensive clinical training. Dental students in Umeå engage in approximately 20 to 24 h of clinical training per week from the third year until their examination. Their initial clinical training occurs during the fourth semester and includes instruction in radiographic examinations. In the fifth semester, they are trained in local anaesthesia, tooth extractions, periodontal treatment, and cariology. The approach of subject-specific education has both advantages and disadvantages. On the positive side, organising education on a subject-by-subject basis allows students to focus and concentrate on specific subjects. However, this approach can be time-consuming, as education in each dental specialty necessitates covering various aspects. Students must acquire knowledge for patient examination, make decisions regarding therapy selection, and assess the prognosis for various treatments. Subject-by-subject education also requires that patients involved in clinical training be evaluated and categorised based on their specific treatment needs, such as cariological or periodontal treatment, before interacting with dental students. In the survey that was distributed, students’ confidence was queried with respect to subject specific clinical tasks. The procedures assessed were formulated in collaboration with the course coordinator for the different clinical subjects, ensuring that students had been exposed to theoretical content, preclinical training, and clinical experience. The results indicate that students rank their confidence quite highly, and there is no significant difference before and after their practical training during the summer break. In the current curriculum at Umeå University, students study both didactic and clinically specific dental subjects throughout their education. This system hinders the students’ comprehensive understanding of dental care, leading to a perception that their advancement in odonatological knowledge is limited when they engage with unselected patients presenting diverse treatment needs. In practice, patient care does not revolve around specific subjects but rather addresses the comprehensive dental care needs of the patient. Assessing students’ confidence in terms of dental responsibility, therapy selection, and follow-up planning may provide a more accurate indication of their preparedness for professional life. In the present study, we registered whether the students had been exposed to the clinical tasks they were supposed to self-assess during their summer clinical internship. They either responded ‘yes’, indicating that they had performed the clinical task during their internship, or ‘no’, indicating that they had not carried out the requested clinical task during their internship. Interestingly, in Survey 2, those who responded with ‘yes’ expressed significant higher confidence in their abilities as a dentist than those who responded with ‘no’ in several of the clinical tasks. The association between experience and knowledge acquisition is well-established. Vosti et al., in their evaluation of medical training in the United States, identified a ‘highly significant correlation between the extent of clinical training and the acquisition and application of clinical knowledge’ [14].

The assessed student group comprised 67 students, and 63 participated in the study. Although we did not specifically examine the influence of gender, it is noteworthy that females constituted two-thirds of the group (45 of 67). There are several studies reporting that more female students than male students reported lower self-confidence in clinical settings [15, 16]. Karaharju-Suvanto et al., who evaluated Finnish dentists to gauge their views on whether undergraduate education adequately prepared them for clinical practice, found a substantial gender difference in the self-assessed competence for completing clinical procedures – i.e., male dentists expressed greater confidence across almost all clinical fields. The authors concluded that more efforts should be made to provide constructive support for dental students with varying approaches to learning clinical skills [17]. One can speculate about the gender perspective when analysing the result in our survey.

The assessment of self-confidence using a ten-point scale, ranging from 0 (not at all confident) to 10 (totally confident), was previously employed by Sjöström and Brundin in the evaluation of various pedagogical approaches during clinical training for local anaesthesia. During the planning phase of this study, we decided to employ the same testing methodology as the ten-step evaluation to assess students’ self-confidence within the context of local anaesthesia education [13]. In a study conducted by Kaur et al., final year BDSc students assessed the attitudes of their peers in the final year of dental studies towards paediatric dentistry training and their confidence treating child patients using a five-step evaluation. The students expressed the highest confidence in administering local anaesthesia to a child. However, their confidence level was rated low when it came to performing pulp therapy procedures on a child. Responses to an open-ended question indicated a preference for additional preclinical training in pulp therapy procedures and managing dental trauma [18].

Umeå students expressed a lack of confidence in the instrumental treatment of caries in children. This can be attributed, in part, to the fact that the curriculum for instrumental treatment of caries in pedodontics is not fully covered until the end of the tenth semester for Umeå students. Additionally, pedodontics may involve emergency endodontic treatment in cases of extensive carious lesions with deep and symptomatic cavities. The self-confidence of Umeå students in performing access preparation for endodontic treatment was found to be low. Using both quantitative and qualitative analyses to investigate anxiety levels of dental students when conducting emergency endodontic treatments, Grock et al. found that these students had varying levels of anxiety, potentially leading to diminished confidence before engaging in emergency endodontic procedures. Based on their findings, the authors suggested restructuring activities to incorporate more practical classes in endodontics [19].

Dealing with dental trauma presents numerous challenges in a clinical setting that involve the medical condition of the affected patient, potential risks of neurological and psychological effects, as well as severe damage to both soft and hard tissues. A thorough examination is essential for establishing a diagnosis, which is the basis for determining the appropriate therapy. Umeå students reported the lowest confidence in handling dento-alveolar trauma. This observation aligns with Sonbol et al.’s findings where students evaluated their confidence using a Visual Analogue Scale during different dental treatments on primary and permanent teeth during practice at paediatric clinics [20]. Dental trauma often affects children and adolescents, and prompt and effective management is of greatest importance for the oral and psychological well-being of individuals experiencing dental trauma [20, 21]. Enhancing students’ self-confidence in handling caries in children, performing endodontic treatments, and managing dental trauma can be achieved by providing dental students with the opportunity to observe a specialist perform the treatments before they encounter these aspects directly. In 2009, Horst et al. emphasised the benefits of such exposure: ‘the opportunity for students to observe and assist before the clinical years of dental school can foster a smoother transition to clinic, enhance learning retention, and improve clinician-patient interactions’ [22]. Dental extraction exhibited a significant increase in self-confidence before and after the summer clinical internship. The oral surgery clinical training programme in Umeå requires students to independently perform operative procedures involving surgical extraction of impacted mandibular third molars. Dental extractions may result in root fractures attributed to root anatomy or dental caries. Patients in northern Sweden often must travel considerable distances to oral and maxillofacial clinics, limiting the option of referrals to specialists. Consequently, the focus during the oral surgery course has been on training surgical removal skills. Ideally, this training instils confidence in students, enabling them to extract teeth independently even when faced with the challenge of removing a fractured root. In this study, we are unable to specify the exact locations where the students completed their clinical placements, other than indicating that they were conducted within the public dental service in Sweden. Additionally, we do not have information regarding the number of procedures performed for each specific task; we can only ascertain whether the procedures were undertaken. This represents a significant limitation of the study. Additional studies involving larger student cohorts are necessary to determine whether the frequency of procedures performed is a significant factor in enhancing self-confidence, or if engaging in a procedure on a limited number of occasions suffices to validate that the knowledge gained during training is adequate for fostering a sense of security in professional practice.

## Conclusion

The outcomes of this survey, focusing on the self-confidence of dental students, suggest that a clinical internship between the ninth and tenth semesters does not significantly enhance students’ confidence in specific dental procedures except for dental extractions. This observation might be attributed to the brevity of clinical training during this period. Alternatively, the gender perspective could have influenced the outcome of the evaluation. The dental programme at Umeå University is presently undergoing a revision, with a new curriculum set to start in January 2025. The updated curriculum aims to incorporate clinical skills training outside the dental school in the later years of education. The findings from this assessment need to be considered when developing the new dental education curriculum at Umeå University. Additional evaluations involving the experiences of the clinical supervisors could potentially contribute to broadening the knowledge of how to optimise clinical training within dental education.

## Data Availability

The data used for this research article can be made available upon request to the authors.
